# A note on factor normalization for deep neural network models

**DOI:** 10.1038/s41598-022-09910-6

**Published:** 2022-04-08

**Authors:** Haobo Qi, Jing Zhou, Hansheng Wang

**Affiliations:** 1 Guanghua School of Management, Peking University, Beijing, 100871 China; 2 Center for Applied Statistics and School of Statistics, Renmin University of China, Beijing, 100872 China

**Keywords:** Engineering, Mathematics and computing

## Abstract

Deep neural network (DNN) models often involve high-dimensional features. In most cases, these high-dimensional features can be decomposed into two parts: a low-dimensional factor and residual features with much-reduced variability and inter-feature correlation. This decomposition has several interesting theoretical implications for DNN training. Based on these implications, we develop a novel *factor normalization* method for better performance. The proposed method leads to a new deep learning model with two important characteristics. First, it allows factor-related feature extraction, and second, it allows for adaptive learning rates for factors and residuals. These model features improve the convergence speed on both training and testing datasets. Multiple empirical experiments are presented to demonstrate the model’s superior performance.

## Introduction

In recent decades, the progress in deep learning, together with advances in graphics processing unit (GPU) devices, has led to the growing popularity of deep neural network (DNN) models in both academia and industry. DNN models have been widely used in various fields for applications such as image classification, speech recognition, and machine translation. However, because of their deep structure, most DNN models are extremely difficult to train. Practical training of a DNN model is often extremely time consuming and highly depends on empirical experience. Therefore, a series of effective optimization methods have been developed for faster DNN training.

According to a recent survey paper^1^, most optimization methods with explicit derivatives can be roughly categorized into two groups: first-order optimization methods and high-order optimization methods. The widely used stochastic gradient descent (SGD) algorithm and its variants^2,3^ are typical examples of first-order optimization methods. The SGD algorithm computes only the first-order derivatives (i.e., gradient) using a randomly sampled batch. Thus, the SGD algorithm can handle large datasets with limited computational resources. Unfortunately, the practical feasibility of SGD comes at the cost of sublinear convergence speed^4^. For better convergence speed, various accelerated SGD algorithms have been developed, for instance, the popularly used momentum method^5,6^ and the Nesterov accelerated gradient descent (NAG)^7,8^ method. Both methods take into consideration information from the previous update gradient direction. Further improvements including AdaGrad^9^, AdaDelta^10^, RMSprop^11^ and Adam^12^ consider element-wise learning rate adjustment, which are known as the adaptive learning rate methods. For a more stable gradient estimation, the stochastic average gradient (SAG)^13^ and stochastic variance reduction gradient (SVRG)^4^ methods have also been developed.

In addition to the first-order optimization methods, high-order optimization methods also exist. Popular examples include Newton’s method and its variants^14,15^. Compared to the first-order methods, high-order methods tend to have faster convergence speed because they consider Hessian matrix information. For example, Newton’s method can achieve a quadratic convergence speed under appropriate conditions^16^. However, calculating and storing the Hessian matrix and its inverse are extremely expensive in terms of both time and storage. This has led to the development of approximation methods, such as the quasi-Newton method^16^ and the stochastic quasi-Newton method^17^. The idea behind these methods is to approximate the inverse Hessian matrix using a positive definite matrix. Popular examples include the Davidon-Fletcher-Powell^18^, Broyden-Fletcher-Goldfarb-Shanno (BFGS)^19,20^, and limited-memory BFGS^21^ methods. Moreover, as a useful technique for achieving fast convergence, various preconditioning techniques are also commonly used^22^. The basic idea of preconditioning is to transform a difficult or ill-conditioned linear system (e.g., $A\theta =b$) into an easier system with better conditions^23^. Consequently, the information contained in the feature covariance can be effectively used^24^. Other interesting methods for extracting useful information from the feature covariance also exist; see, for example,^25,26^, and^27^.

However, to the best of our knowledge, no existing models or methods are specifically designed for high-dimensional features with a factor structure. In the meanwhile, ample empirical experience suggests that high-dimensional features usually demonstrate a strong factor-type covariance structure^28–30^. In other words, a significant amount of feature variability can be explained by a latent factor with very low dimensions. Consequently, the original features can be decomposed into two parts: a low-dimensional factor that accounts for a significant portion of the total volatility, and a residual part with the factor effects removed. This residual part has the same dimensions as the original feature. Consequently, the variability is significantly reduced. Moreover, the inter-feature correlation is also substantially reduced. To this end, the original learning problem concerning the high-dimensional features can be decomposed into two learning subproblems. The first is related to latent factors. This is relatively simple because the factor dimensions are very low. The second problem is related to the residual feature. Unfortunately, this remains a challenging problem because of the feature’s high dimensions. However, compared with the original problem, this modified problem is much easier to solve because the inter-feature dependence is substantially reduced. For practical implementation, we propose here a novel method called factor normalization. It begins with a benchmark model (e.g., AlexNet or ResNet50) and then slightly modifies it to create a new model structure. Unlike the benchmark model, the new model takes the latent factor and residuals as different inputs. The original structure is retained to process the residuals, and the latent factor is then returned to the modified model in the last layer. This compensates for the information loss caused by factor extraction. Thus, the new model allows factor-and residual-related features to be processed separately. Furthermore, different (i.e., adaptive) learning rates can be allowed for the factor and residuals. This leads to adaptive learning and thus a fast convergence speed.

The remainder of this article is organized as follows. Section “theoretical motivation” develops our theoretical motivation and provides statistical insights. Section “proposed method” details the proposed model. Section “experiments” demonstrates the outstanding performance of the proposed model through extensive empirical experiments, and Section “conclusion” concludes the article with a brief discussion of future research. All detailed techniques are relegated to the “Appendix”.

## Theoretical motivation

We developed our new model based on several interesting theoretical motivations derived from different perspectives. Because the SGD algorithm is a stochastic version of the gradient descent (GD) algorithm, we focus on the standard GD algorithm in this section for simplicity.

### GD algorithm

Let $(X_i,Y_i)$ be the observation collected from the *i*th instance with $1\le i\le N$, where $Y_{i}$ is often the class label and $X_i = (X_{i1}, \ldots ,X_{ip})^\top \in \mathbb {R}^p$ is an associated *p*-dimensional feature. The loss function, evaluated at *i*, is defined as $\ell (Y_i, X^\top _i\theta )$, where $\theta \in \mathbb {R}^p$ is an unknown parameter. The global loss is then given by ${\mathscr {L}}_{N}(\theta ) = N^{-1}\sum ^N_{i=1}\ell (Y_i, X^\top _i\theta )$. The global gradient is given by $\dot{{\mathscr {L}}}_{N}(\theta )=N^{-1}\sum _{i=1}^{N}{\dot{\ell }}(Y_{i},X_{i}^\top \theta )X_{i}$ and ${\dot{\ell }}(y,z)=\partial \ell (y,z)/\partial z$. Let $\hat{\theta }^{(t)}$ be the estimator obtained at the *t*th iteration. Subsequently, the GD algorithm updates the parameter as $\hat{\theta }^{(t+1)} = \hat{\theta }^{(t)} - \alpha \dot{{\mathscr {L}}}_{N}(\hat{\theta }^{(t)})$. Here, $\alpha$ is a scalar referred to as the learning rate^2^. Assume that ${\mathscr {L}}_{N}(\hat{\theta }^{(t)})$ reaches its minimum at $\hat{\theta }$, such that $\dot{{\mathscr {L}}}_{N}(\hat{\theta }) = 0$. Subsequently, we apply the Taylor expansion for $\dot{{\mathscr {L}}}_{N}(\theta )$ at $\hat{\theta }$. This leads to

$$\begin{aligned} \hat{\theta }^{(t+1)}- \hat{\theta }= & {} \Big \{I_p - \alpha \ddot{{\mathscr {L}}}_{N}(\hat{\theta }) \Big \}(\hat{\theta }^{(t)} - \hat{\theta }) + o(\Vert \hat{\theta }^{(t)} - \hat{\theta }\Vert ^2)\\= & {} {\mathbb {K}} (\hat{\theta }^{(t)} - \hat{\theta }) + o(\Vert \hat{\theta }^{(t)} - \hat{\theta }\Vert ^2), \end{aligned}$$

where ${\mathbb {K}} = I_p - \alpha \ddot{{\mathscr {L}}}_{N}(\hat{\theta })$. We refer to ${\mathbb {K}}$ as a *contraction operator*, which plays a very important role in optimization. Intuitively, all the eigenvalues of ${\mathbb {K}}$ should lie within $(-1,1)$. Otherwise, the algorithm might not numerically converge.

#### Proposition 1

Assume $\ddot{{\mathscr {L}}}_{N}(\hat{\theta })$ is a positive definite matrix. Let $\lambda _1\ge \lambda _2\ge  \cdots \ge \lambda _p> 0$ be the eigenvalues of $\ddot{{\mathscr {L}}}_{N}(\hat{\theta })$. To converge the GD algorithm, we must have $0<\alpha <1/\lambda _1$.

### Condition number

From Proposition 1, we know that the learning rate cannot be too large. Otherwise, the GD algorithm may not numerically converge. The size of the learning rate is controlled by the largest eigenvalue of the Hessian matrix, $\ddot{{\mathscr {L}}}_{N}({\hat{\theta }})$. The larger the value of $\lambda _{1}$, the smaller the learning rate and the slower the convergence speed. This problem is particularly serious if the condition number (i.e., $\lambda _{1}/\lambda _{p}$) of the Hessian matrix is very large. In this case, a large $\lambda _{1}$ value forces the learning rate, $\alpha$, to be very small. Meanwhile, other small eigenvalues ($\lambda _{j}$ for $j\ne 1$) reduce the convergence speed along the corresponding eigendirections. Thus, practitioners should want the condition number of the Hessian matrix to be as small as possible. However, as mentioned previously, most high-dimensional features have a strong factor structure. In other words, the size of the top eigenvalues of covariance matrix $\Sigma$ is typically much larger than that of the rest. Consequently, the condition number of the covariance matrix is typically very large. Therefore, it is of great interest to investigate how this factor structure would affect the condition number of the Hessian matrix.

To address this important problem, we evaluate the expected Hessian matrix as ${\mathbb {H}} = E(\ddot{{\mathscr {L}}}(\theta )) = E\{ \ddot{\ell }(Y_{i},X_{i}^\top \theta )X_{i}X_{i}^\top \}$. where $\ddot{\ell }(y,z)=\partial {\dot{\ell }}(y,z)/\partial z$ denotes the second-order derivative of $\ell (y,z)$ with respect to *z*. For illustrative purposes: assume that $X_{i}$ is normally distributed, with a mean of zero. Recall that the covariance matrix is $\Sigma$, so define ${\tilde{X}}_{i} = \Sigma ^{-1/2}X_{i}$ and ${\tilde{\theta }} = \Sigma ^{1/2}\theta$. We then rewrite ${\mathbb {H}}$ as ${\mathbb {H}} = \Sigma ^{1/2}E\{\ddot{\ell }(Y_{i},{\tilde{X}}_{i}^\top {\tilde{\theta }}){\tilde{X}}_{i}{\tilde{X}}_{i}^\top \}\Sigma ^{1/2} = \Sigma ^{1/2}\widetilde{{\mathbb {H}}} \Sigma ^{1/2}$, where $\widetilde{{\mathbb {H}}} = E\{\ddot{\ell }(Y_{i},{\tilde{X}}_{i}^\top {\tilde{\theta }}){\tilde{X}}_{i}{\tilde{X}}_{i}^\top \}$. Let *A* be an arbitrary positive-definite matrix, and define $\lambda _{\text{ max }}(A)$ and $\lambda _{\text{ min }}(A)$ as the maximum and minimum eigenvalues of *A*, respectively. We then have $\lambda _{\text{ max }}({{\mathbb {H}}}) \ge \lambda _{\text{ max }}(\Sigma )\lambda _{\text{ min }}(\widetilde{{\mathbb {H}}})$ and $\lambda _{\text{ min }}({{\mathbb {H}}}) \le \lambda _{\text{ min }}(\Sigma )\lambda _{\text{ max }}(\widetilde{{\mathbb {H}}})$. This further suggests that the condition number of ${{\mathbb {H}}}$ (i.e., ${\text {con}}({\mathbb {H}})=\lambda _{\text{ max }}({{\mathbb {H}}})/\lambda _{\text{ min }}({{\mathbb {H}}})$) satisfies the following inequality:

1 $$\begin{aligned} {\text {con}}({\mathbb {H}})\ge {\text {con}}(\Sigma )/{\text {con}}(\widetilde{{\mathbb {H}}}), \end{aligned}$$

where ${\text {con}}(A)$ stands for the condition number of an arbitrary positive-definite matrix. Note that $\widetilde{{\mathbb {H}}}$ is the expected Hessian matrix under an extremely ideal situation, where the input feature follows a standard multivariate normal distribution. This is arguably the ideal situation for numerical optimization. Thus, we can reasonably expect that ${\text {con}}(\widetilde{{\mathbb {H}}})$ will not be very large in this case. Moreover, from (1), we know that ${\text {con}}(\Sigma )$ should play an important role in determining ${\text {con}}({{\mathbb {H}}})$. That is, the condition number of $\Sigma$ is affected by ${\text {con}}({{\mathbb {H}}})$. This makes numerical optimization by a standard GD algorithm extremely difficult and thus calls for a novel solution.

### Factor linear subspace

To further explain the motivation behind the proposed method, we provide a theoretical justification from a different perspective. Assume a standard factor model as $X_i = BZ_i + {\mathscr {E}}_{i}$, where $Z_i\in \mathbb {R}^d$ is a vector with low dimensionality (i.e., $d\ll p$) and $B \in \mathbb {R}^{p\times d}$ is the corresponding factor loading matrix with $d\ll p$. Consider the general loss function ${\mathscr {L}}(\theta )$, and recall that the global gradient is $\dot{{\mathscr {L}}}_{N}(\theta ) = N^{-1}\sum ^N_{i=1}\dot{\ell }(Y_i, X^\top _i\theta ) X_i$. We then have $\dot{{\mathscr {L}}}_{N}(\theta ) = Q_{1}+Q_{2}$, where $Q_{1} = B N^{-1}\sum ^N_{i=1}\dot{\ell }(Y_i, X^\top _i\theta )Z_i\in {\mathscr {S}}(B)$, $Q_{2} = N^{-1}\sum ^N_{i=1}\dot{\ell }(Y_i, X^\top _i\theta ) \varepsilon _i$, and ${\mathscr {S}}(B)$ denotes the linear subspace spanned by the column vectors of *B*. The covariance structure of the global gradient can then be written as ${\text {cov}}\{\dot{{\mathscr {L}}}_{N}(\theta )\} = {\text {cov}}(Q_1) + {\text {cov}}(Q_2),$ where ${\text {cov}}(Q_1) = B\Sigma _z B^\top /N$ , $\Sigma _z = {\text {cov}}\{\dot{{\mathscr {L}}}(Y_i, X^\top _i\theta )Z_i\}\in \mathbb {R}^{d\times d}$, ${\text {cov}}(Q_2) = \Sigma _{\varepsilon }/N$, and $\Sigma _{\varepsilon } = {\text {cov}}\{\dot{{\mathscr {L}}}(Y_i, X^\top _i\theta )\varepsilon _i\}\in \mathbb {R}^{d\times d}$. It is then of interest to study the relative sizes of cov$(Q_{1})$ and cov$(Q_{2})$ under appropriate metrics.

#### Proposition 2

Assume there exists constant $0< \tau _{\text {min}}< \tau _{\text {max}}< \infty$, such that $\tau _{\text {min}}<\lambda _{\text {min}}(\Sigma _z) \le \lambda _{\text {max}}(\Sigma _z) < \tau _{\text {max}}$ and $\tau _{\text {min}}<\lambda _{\text {min}}(\Sigma _{\varepsilon }) \le \lambda _{\text {max}}(\Sigma _{\varepsilon }) < \tau _{\text {max}}$. Furthermore, assume that $\lambda _{\text {min}}(B^\top B/p)>\tau _{\text {min}}$. We then have ${\text {tr}}\{{\text {cov}}(Q_1)\}/{\text {tr}}\{ {\text {cov}}(Q_2)\}\ge \tau ^2_{\text {min}}/\tau _{\text {max}}$.

For most DNN models, the parameter dimension, *p*, is very high. This makes the model structure sufficiently flexible. Accordingly, one might expect the associated GD (or SGD) algorithm should search the entire high- dimensional parameter space (e.g., $\mathbb {R}^{p}$) in a very flexible way. However, the above proposition indicates that the estimated gradient direction is not as flexible as we might expect. In contrast, it always has a significant portion (i.e., $Q_1$) trapped in a very low-dimensional linear subspace, ${\mathscr {S}}(B)$. This brings a positive effect. Because the linear subspace, ${\mathscr {S}}(B)$, has a very low dimension (i.e., *d*), the overfitting effect caused by to $Q_1$ becomes negligible. However, this positive effect also comes at a convergence cost. By trapping a significant portion of the gradient direction in ${\mathscr {S}}(B)$, the ability of a GD algorithm to explore directions other than ${\mathscr {S}}(B)$ is considerably sacrificed. This problem can be solved if the directions of the factor (i.e., $Q_1$) and the residual features (i.e., $Q_2$) can be treated separately. This is another important theoretical insight that drove us to develop our new model.

## Proposed method

Inspired by the theoretical findings discussed in the previous section, we propose a *factor normalization* method. This new method is specifically designed for deep models with high-dimensional features and factor structures. It begins with an important benchmark model (e.g., AlexNet or ResNet50), and can be implemented in three steps: factor decomposition, model reconstruction, and adaptive learning. The details of which are discussed in the following subsections.

### Factor decomposition

In the first step, we conduct a standard singular value decomposition for the high-dimensional features. By doing so, a low-dimensional factor can be estimated. Accordingly, the original features can be decomposed into two parts. The first is related to the factor and is referred to as the factor part. The second comprises the original features, but the factor effects are removed. This part is referred to as the residual part (or residual feature). Specifically, during the training process, we assume that the centralized input matrix is written as $X = (X_1 \ldots ,X_{N_0})^\top \in \mathbb {R}^{N_0\times p}$, with $n=\min (N_0,p)$, such that the column mean of *X* is 0. We then conduct a singular value decomposition as $X = U\Lambda V^\top = \sum ^n_{i=1}\lambda _iu_iv^\top _i$, where $U = (u_1 \ldots ,u_n)\in \mathbb {R}^{N_0\times n}$ and $V=(v_1,v_n)\in \mathbb {R}^{p\times n}$, with $U^\top U = V^\top V = I_{n}$ and $I_{n}\in \mathbb {R}^{n \times n}$ representing the identity matrix. Moreover, $\Lambda = \text {diag}(\lambda _1 \ldots ,\lambda _n)$ is a diagonal matrix, with $\lambda _1\ge \lambda _2\ge  \cdots \ge \lambda _n\ge 0$. We define $V_d = (v_1 \ldots ,v_d)$ as a truncated orthogonal matrix that collects the top *d* singular vectors of *V*. Then, the latent factors can be estimated as $\hat{Z} = X V_d$, where $\hat{Z}\in \mathbb {R}^{N_0\times d}$ denotes the estimated factor part. Finally, we use linear regression to estimate the residual feature matrix as $\hat{{\mathscr {E}}} = X - \hat{Z}\hat{B}$, where $\hat{B} = (\hat{Z}^\top \hat{Z})^{-1} \hat{Z}^\top X\in \mathbb {R}^{d\times p}$ denotes the estimated factor-loading matrix. Thus, the original feature matrix, *X*, is decomposed into two parts: the factor part, ${\hat{Z}}$, and the residual part, $\hat{{\mathscr {E}}}$. For the testing process, we first use the column mean calculated from the training data to centralize the testing data. We assume that the centralized testing input matrix is written as ${\widetilde{X}} = ({\widetilde{X}}_1 \ldots ,{\widetilde{X}}_{N_1})^\top \in \mathbb {R}^{N_1\times p}$. Then, we use the singular vectors, $V_d$, and regression coefficients, $\widehat{B}$, calculated from the training data to decompose the testing data into factor part ${\widetilde{Z}}$ and residual part $\widetilde{{\mathscr {E}}}$ as ${\widetilde{Z}} = {\widetilde{X}}V_d$ and $\widetilde{{\mathscr {E}}} = {\widetilde{X}} - {\widetilde{Z}}\hat{B}$.

### Model reconstruction

In the second step, we modify a given benchmark model (e.g., ResNet50) into a new model for better performance. More specifically, consider a standard DNN model with (1) an input layer with original feature *X*; (2) an output layer, which often has a dense structure; and (3) sophisticated latent layers between the input and output layers. We then replace the original feature of the input layer (i.e., *X*) with the residual feature (i.e., $\hat{{\mathscr {E}}}$). Next, the residual feature is fully processed by sophisticated latent layers until it reaches the output layer. Before the DNN model constructs the last dense layer, we return the estimated latent factor (i.e., ${\hat{Z}}$) to the output layer to compensate for the information loss due to factor extraction.

**Figure 1:**
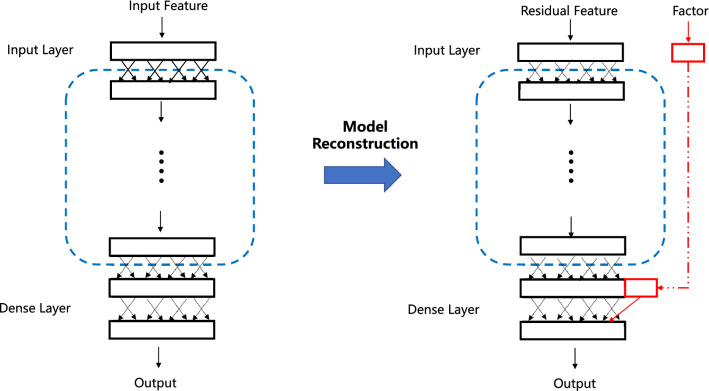
Graphic illustration of model reconstruction for factor normalization. The rectangles represent the DNN feature maps. The blue dashed rectangle represents the latent layers between the input and the last dense layer. The left panel shows the original DNN structure with the original input feature. The right panel shows the modified DNN structure with factor and residual features. As can be seen, the factor and residual features are treated separately. The latent factor is reinserted back into the model before the last dense output layer.

This leads to a new model structure with two interesting characteristics. The high-dimensional residual term is still processed using a sophisticated benchmark model. However, the low-dimensional latent factor is not processed in the same manner. This interesting structure is particularly desirable for the following reasons. First, in most cases, we find that a one-dimensional factor is already sufficient. In this case, the latent factor is univariate. As such, any nonlinear transformation (e.g., from using a DNN) should be only minimally different from the original factor in terms of the information contained. Thus, there is little need for further sophisticated nonlinear transformation of the univariate latent factor. In contrast, the residual form remains high dimensional. However, sophisticated nonlinear transformation is still very useful for high-quality feature extraction. Second, to achieve adaptive learning, and thus better performance, we want to use different learning rates for the factor and residuals. If the factor is placed in an intermediate layer, the practical implementation of adaptive learning using TensorFlow programming could be extremely and unnecessarily complicated. This explains why the latent factor is set as the input for the last layer. Figure 1 provides a more intuitive visual explanation of the new model structure.

### Adaptive learning

As previously discussed , with this modified model, the convergence speed remains very slow if we adopt a standard SGD algorithm. For a reliable convergence, a smaller learning rate should be used for the parameters associated with the factor because the factor variability is large. In contrast, much larger learning rates should be used for the parameters associated with the residual features. Otherwise, their limited variability could cause the convergence speed to be very slow. This suggests that adaptive (i.e., different) learning rates should be used for the factor and residuals. Assume that the learning rate used in a standard SGD is $\alpha$. Recall that in Subsection 3.1, we assume that $\lambda _j$ is the *j*-th largest singular value calculated on training input matrix *X*. Then, the factor-associated learning rate is set as $\alpha _j = \alpha \lambda ^2_1/\lambda ^2_j$ for $j=1 \ldots ,d$. In contrast, the residual-associated learning rate is set the same as $\alpha _{\varepsilon }=\alpha \lambda ^2_{1}/\lambda ^2_{d+1}$. Consequently, the learning rates used for the latent factor become much smaller than those of the residual features. It should be noted that the adaptive learning rates used here are different from those used by AdaGrad or Adam^9,12^. The AdaGrad and Adam methods adjust their learning rates dynamically for each iteration and adaptively for each dimension during the training process.

## Experiments

To empirically demonstrate the proposed factor normalization (FN) model, we conducted various experiments using different models, including logistic regression, multilayer fully connected neural networks, and deep convolutional neural networks. The experiments were run on a Tesla P100 GPU with 16 GB memory.

### Logistic regression

**Figure 2:**
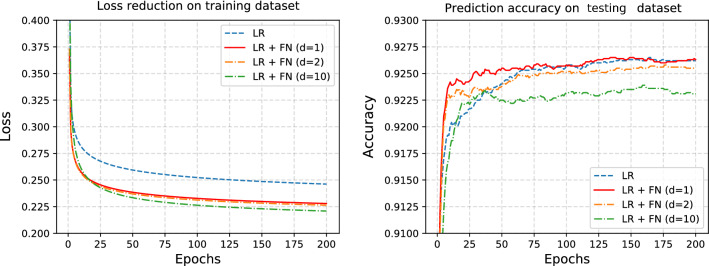
LR stands for logistic regression and FN stands for factor normalization. The left panel shows the training loss of the logistic regression model and FN model optimized by SGD on MNIST images. The right panel shows the prediction accuracy on the testing dataset. Different numbers of factors are considered (i.e., $d=1,2,10$).

We begin with a simple logistic regression (LR) model and evaluate the proposed model using the MNIST dataset. This is a classification task with ten classes, 60,000 instances for training, and 10,000 instances for testing. The input feature is a $28\times 28$ pixel matrix. To implement the logistic regression, we reshape the input feature into a 784-dimensional vector. Subsequently, FN models with various factor dimensions ($d = 1,2,10$) are used to reconstruct the baseline model. The SGD optimizer is used to optimize both the baseline and FN models. Detailed results are presented in Fig. 2. For comparison purposes, all models are trained for many epochs so that their performance on the testing data is fully converged. Specifically, a batch size of 200 with 200 epochs is adopted and we use constant learning rate 0.05 during training process. The left panel of Fig. 2 shows the training loss for the various models. We find that the FN models consistently outperform their benchmark counterparts, because the loss curves in the FN models are always below the baseline model. The right panel of Fig. 2 shows the accuracy of the results. We can see that the accuracy curves of the FN models reach a plateau earlier than the baseline model. Additionally, there appears to be little difference in the performance of the FN models with different factor dimensions *d*. Therefore, we fix $d=1$ for the subsequent experiments.

### Multilayer neural networks

**Figure 3:**
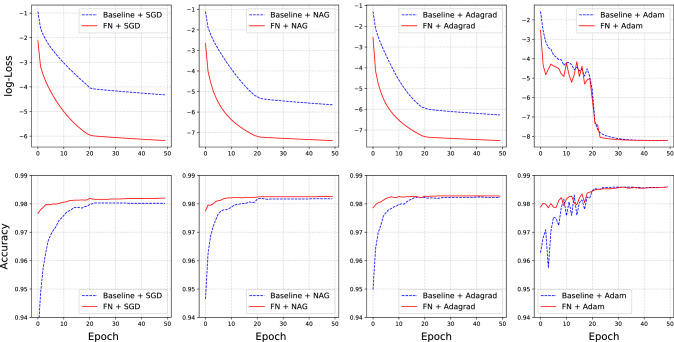
Here, the top panel is the training loss in log scale for the baseline model (blue dash line) and FN model (red solid line) with four different optimizers. The bottom panel shows the testing accuracy for the baseline model (blue dashed line) and the FN model (red solid line) with different optimizers.

Next, we consider a more complicated multilayer neural network model. The proposed model has two fully connected hidden layers with 1,000 neurons. Rectified linear unit (ReLU) transformation is used in each hidden layer for activation^12^. The settings of the experiment are almost the same as those in Subsection 4.1, except for the following differences. First, we only consider the FN model in which $d=1$. Second, for a comprehensive comparison, we consider four different optimizers: SGD, NAG, AdaGrad, and Adam. This results in four groups of comparative experiments. Here, the baseline model is an original DNN model with a specific optimization method. It is compared to an FN model, which is reconstructed from the same original model using the same optimization setting. Furthermore, we train all models for a total of 50 epochs. For each optimizer, the initial learning rate is chosen by grid search and decays by 0.2 after 20 epochs. The detailed results are presented in Fig. 3.

The top panel of Fig. 3 shows the training loss, in log scale, for the four optimization methods on the baseline and FN models. We find that for each optimizer, the FN model always achieves a smaller training loss than the baseline model. This difference is more obvious in the cases of SGD, NAG, and AdaGrad. The bottom panel of Fig. 3 reports the prediction accuracy of the testing dataset. We find that for each optimizer, the accuracy of the FN model is higher than that of the baseline model.

### Convolutional neural networks

Convolutional neural networks (CNNs) are the most popular models used in image classification and object detection tasks. The main difference between CNNs and multilayer neural networks is that CNNs contain many convolutional and pooling layers, which provide efficient feature extraction and lead to excellent prediction performance. However, CNNs typically have complicated model structures with a large number of parameters. Here, we consider two classical CNN models: AlexNet^31^ and ResNet50^32^. It should be noted that the CNN model considered here typically has a a fully connected layer at the end. This is because the proposed FN model always adds the factors to the last dense layer. The datasets used in this study are CIFAR10^33^ and CatDog released by Kaggle in 2013.

**Figure 4:**
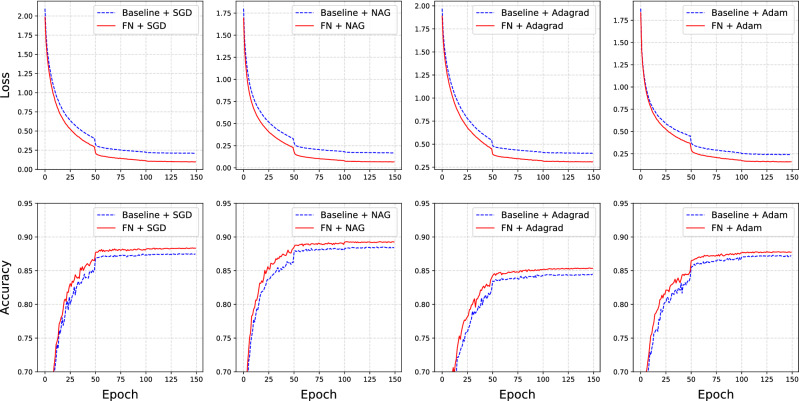
Results of training AlexNet on the CIFAR10 dataset. The top panel shows the training loss of the baseline (blue dashed line) and FN models (red solid line) with four different optimizers. The bottom panel shows the testing accuracy of the baseline (blue dashed line) and FN models (red solid line) with different optimizers. The curves represent the median training loss and testing accuracy of the ten repeated experiments.

**Figure 5:**
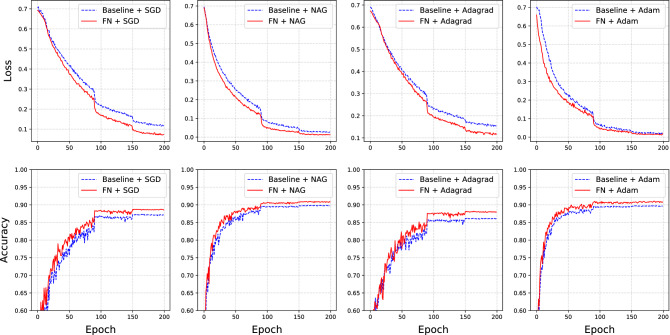
Results of training ResNet50 on CatDog dataset. The top panel shows the training loss of the baseline (blue dashed line) and FN models (red solid line) with four different optimizers. The bottom panel shows the testing accuracy of the baseline (blue dashed line) and FN models (red solid line) with different optimizers. The curves represent the median training loss and testing accuracy of ten repeated experiments.

Similar to the experiment described in Subsection 4.2, we evaluate four different optimizers: SGD^2^, NAG^34^, AdaGrad^9^, and Adam^12^. For the AlexNet experiments on the CIFAR10 dataset, we train the model for a total of 150 epochs, and the batch size is set to 128. The learning rate for each optimizer is selected by a grid search, and decays by 0.2 at the 50th and 100th epochs. For the ResNet50 experiments on the CatDog dataset, we train the model for 200 epochs, and the batch size is set to 128. Similarly, the initial learning rates are determined by a grid search, and decay by 0.2 at the 90th and 150th epochs. To achieve stable performance, we repeat each experiment ten times. Then, we draw the loss/accuracy curves for both the FN and the baseline models using median statistics. For illustration, we summarize the results of AlexNet on CIFAR10 and ResNet50 on CatDog in Figs. 4 and 5, respectively.

From these figures, we find that the FN models have a relatively lower loss than the baseline models during training. Furthermore, compared to the baseline models, the FN models reach the same prediction accuracy level in fewer epochs. In addition, the final prediction accuracy of the FN models on the testing set is slightly higher (approximately 1–2%) than that of the baseline models. Consequently, the time required for the FN models to reach optimal baseline prediction accuracy is less than that required by the baseline models. It is worth noting that the time required to conduct SVD in the FN model is preprocessed before training, which is significantly less than the training cost. In summary, we found that the FN model can improve the performance of the baseline model in terms of time cost, training loss, and prediction accuracy. Similar conclusions can be drawn from the results for ResNet50 on CatDog in Fig. 5.

## Conclusion

In this paper, we propose a novel *factor normalization* method for fast DNN training. This concept was inspired by the fact that many DNN models involve high-dimensional features, and these features often exhibit a strong factor structure. The proposed method has three key components. First, it decomposes high-dimensional input features into two parts: a factor part with low dimensionality and a residual part. Second, it slightly modifies a given DNN model so that the effects of the latent factor and residual features can be processed separately. Last, to train a modified DNN model, a new SGD algorithm is developed. This allows for adaptive learning rates for the factor and residual parts.

To conclude this article, we present here a number of interesting topics for future study. First, our current FN model performs factor decomposition on the input features. Even if different components of the input feature are completely independent of each other, the resulting feature map after multiple convolutional layers might still have a strong factor structure. Therefore, it is worth studying whether factor decomposition should be conducted for feature maps. Second, the current FN model does not insert the estimated factors back into the DNN models until the last layer. As such, determining what happens if the estimated factors are inserted into earlier layers is another problem of great interest.

## Supplementary Information

Supplementary Information.
