# Supplementary material for: A note on factor normalization for deep neural network models
Source: Sci Rep. 2022 Apr 8;12:5909. doi: 10.1038/s41598-022-09910-6 (PMC8993835; doi:10.1038/s41598-022-09910-6)
Supplement: Supplementary file 1 — Supplementary Information. [file 41598_2022_9910_MOESM1_ESM.pdf]

A Note on Factor Normalization for Deep Neural Network Models

Haobo Qi[1], Jing Zhou[2,*], and Hansheng Wang[1]

[1]Guanghua School of Management, Peking University, Beijing, 100871, China
[2]Center for Applied Statistics and School of Statistics, Renmin University of China, Beijing, 100872, China
[*]jing.zhou@ruc.edu.cn

ABSTRACT

Deep neural network (DNN) models often involve features of high dimensions. In most cases, the high-dimensional features can be decomposed into two parts. The first part is a low-dimensional factor. The second part is the residual feature, with much-reduced variability and inter-feature correlation. This leads to a number of interesting theoretical findings for deep neural network training. Accordingly, we are inspired to develop a new *factor normalization* method for better performance. The proposed method leads to a new deep learning model with two important features. First, it allows factor related feature extraction. Second, it allows adaptive learning rates for factors and residuals, respectively. This leads to fast convergence speed on both training and validation datasets. A number of empirical experiments are presented to demonstrate its superior performance.

APPENDIX

Proof of proposition 1

Definition 1. *A function $f : D \subset \mathbb{R}^p \mapsto \mathbb{R}$ is called L-smooth if its gradient $\dot{f}$ satisfies the Lipschitz continuous condition with the Lipschitz constant L.*

Lemma 1. *By (1), If function $f : D \subset \mathbb{R}^p \mapsto \mathbb{R}$ is L-smooth, then for $x, y \in D$,*

$$|f(x) - f(y) - \langle \dot{f}(y), x - y \rangle| \leq \frac{L}{2}\|x - y\|^2.$$

Here, $\langle x, y\rangle$ denotes the inner product of two arbitrary vectors x, y with the same dimension.

Lemma 2. *Assume that the global loss function $\mathscr{L}_N(\theta) : D \subset \mathbb{R}^p \mapsto \mathbb{R}$ is L-smooth, convex and has a unique minimum at $\hat{\theta}$. Then, for any $\hat{\theta}^{(t)} \in D, t = 0, 1 \ldots, T$, we have*

$$\mathscr{L}_N(\hat{\theta}^{(t)}) - \mathscr{L}_N(\hat{\theta}) \leq \left\langle \dot{\mathscr{L}}_N(\hat{\theta}^{(t)}), \hat{\theta}^{(t)} - \hat{\theta} \right\rangle - \frac{1}{2L}\left\| \dot{\mathscr{L}}_N(\hat{\theta}^{(t)}) \right\|^2.$$

Proof: Consider a point $z = \hat{\theta} + \frac{1}{L}\dot{\mathscr{L}}_N(\hat{\theta^{(t)}})$. On the one hand, since $\mathscr{L}_N(\theta)$ is convex,

$$\begin{aligned}\mathscr{L}_N(\hat{\theta}^{(t)}) - \mathscr{L}_N(z) &\leq \left\langle \dot{\mathscr{L}}_N(\hat{\theta}^{(t)}), \hat{\theta}^{(t)} - z \right\rangle \\ &= \left\langle \dot{\mathscr{L}}_N(\hat{\theta}^{(t)})\hat{\theta}^{(t)} - \hat{\theta} \right\rangle + \left\langle \dot{\mathscr{L}}_N(\hat{\theta}^{(t)}), \hat{\theta} - z \right\rangle.\end{aligned}$$

However, since $\mathscr{L}_N(\theta)$ is L-smooth and according to Lemma 1, we have

$$\mathscr{L}_N(z) - \mathscr{L}_N(\hat{\theta}) \leq \langle \dot{\mathscr{L}}_N(\hat{\theta}), z - \hat{\theta} \rangle + \frac{L}{2}\|z - \hat{\theta}\|^2 = \frac{L}{2}\|z - \hat{\theta}\|^2.$$

Then,

$$\begin{aligned}\mathscr{L}_N(\hat{\theta}^{(t)}) - \mathscr{L}_N(\hat{\theta}) &= \mathscr{L}_N(\hat{\theta}^{(t)}) - \mathscr{L}_N(z) + \mathscr{L}_N(z) - \mathscr{L}_N(\hat{\theta}) \\ &\leq \left\langle \dot{\mathscr{L}}_N(\hat{\theta}^{(t)}), \hat{\theta}^{(t)} - \hat{\theta} \right\rangle + \left\langle \dot{\mathscr{L}}_N(\hat{\theta}^{(t)}), \hat{\theta} - z \right\rangle + \frac{L}{2}\|z - \hat{\theta}\|^2 \\ &= \left\langle \dot{\mathscr{L}}_N(\hat{\theta}^{(t)}), \hat{\theta}^{(t)} - \hat{\theta} \right\rangle - \frac{1}{2L}\left\| \dot{\mathscr{L}}_N(\hat{\theta}^{(t)}) \right\|^2.\end{aligned}$$

Q.E.D

Assume that $\hat{\theta}^{(t+1)}$ and $\hat{\theta}^{(t)}$ are solutions after the $(t+1)$-th and t-th iterations, respectively. Then,

$$\begin{aligned}\left\|\hat{\theta}^{(t+1)}-\hat{\theta}\right\|^2 =&\left\|\hat{\theta}^{(t)}-\alpha\dot{\mathscr{L}}_N(\hat{\theta}^{(t)})-\hat{\theta}\right\|^2\\ =&\left\|\hat{\theta}^{(t)}-\hat{\theta}\right\|^2-2\alpha\left\langle\dot{\mathscr{L}}_N(\hat{\theta}^{(t)}),\hat{\theta}^{(t)}-\hat{\theta}\right\rangle\\ &+\alpha^2\left\|\dot{\mathscr{L}}_N(\hat{\theta^{(t)}})\right\|^2.\end{aligned}$$

According to Lemma 2 and note that $\mathscr{L}_N(\hat{\theta}^{(t)})\geq\mathscr{L}_N(\hat{\theta})$, we have

$$0\leq\mathscr{L}_N(\hat{\theta}^{(t)})-\mathscr{L}_N(\hat{\theta})\leq\left\langle\dot{\mathscr{L}}_N(\hat{\theta}^{(t)}),\hat{\theta}^{(t)}-\hat{\theta}\right\rangle-\frac{1}{2L}\left\|\dot{\mathscr{L}}_N(\hat{\theta}^{(t)})\right\|^2.$$

which implies

$$-\left\langle\dot{\mathscr{L}}_N(\hat{\theta}^{(t)}),\hat{\theta}^{(t)}-\hat{\theta}\right\rangle\leq-\frac{1}{2L}\left\|\dot{\mathscr{L}}_N(\hat{\theta}^{(t)})\right\|^2.$$

Substituting this inequality into equation (1), we have

$$\left\|\hat{\theta}^{(t+1)}-\hat{\theta}\right\|^2\leq\left\|\hat{\theta}^{(t)}-\hat{\theta}\right\|^2-\alpha\left(\frac{1}{L}-\alpha\right)\left\|\dot{\mathscr{L}}_N(\hat{\theta}^{(t)})\right\|^2. \tag{A.1}$$

Equation (2) gives the convergence condition of the gradient decent algorithm. As long as $\alpha\leq1/L$, we have $\left\|\hat{\theta}^{(t+1)}-\hat{\theta}\right\|^2\leq\left\|\hat{\theta}^{(t)}-\hat{\theta}\right\|^2$. Note that we only consider the first -order expansion of $\mathscr{L}_N(\theta)$ at point $\hat{\theta}$ in the proof of Lemma 2. Then, the Lipschitz constant L can be substituted by the largest eigenvalue of $\ddot{\mathscr{L}}_N(\hat{\theta})$, that is, λ_1. As a result, the convergence condition can be written as $\alpha\leq1/\lambda_1$.

Proof of Proposition 2

On the one hand, we can verify that

$$\begin{aligned}\operatorname{tr}(\operatorname{cov}(Q_1)) =&\operatorname{tr}\left(\frac{B\Sigma_z B^\top}{N}\right)\geq\tau_{\min}\operatorname{tr}\left(\frac{BB^\top}{N}\right)\\ =&p\tau_{\min}\operatorname{tr}\left(\frac{BB^\top}{pN}\right)\geq\frac{\tau_{\min}^2 p}{N}.\end{aligned}$$

On the other hand, we have

$$\operatorname{tr}(\operatorname{Cov}(Q_2))=N^{-1}\operatorname{tr}(\Sigma_\varepsilon)\leq\frac{p}{N}\tau_{\max}.$$

As a result, we have $\operatorname{tr}\{\operatorname{cov}(Q_1)\}/\operatorname{tr}\{\operatorname{cov}(Q_2)\}\geq\tau_{\min}^2/\tau_{\max}$.

References

1. Boyd, Vandenberghe & Faybusovich. Convex optimization. *IEEE Transactions on Autom. Control.* **51**, 1859–1859 (2006).
